# Contribution of Interleukin-10-592 (-590, -597) C>A Polymorphisms to Periodontitis Susceptibility: An Updated Meta-Analysis Based on 18 Case-Control Studies

**DOI:** 10.1155/2018/2645963

**Published:** 2018-09-19

**Authors:** Yao Li, Ge Feng, Yuejia Deng, Jinglin Song

**Affiliations:** Department of Periodontology, Stomatological College, Chongqing Medical University, Chongqing 400016, China

## Abstract

**Introduction:**

The association between interleukin-10- (IL-10-) 592 (-590, -597) C>A polymorphisms and susceptibility to chronic or aggressive periodontitis (CP or AgP) is conflicting. This meta-analysis is aimed at quantitatively estimating the association.

**Materials and Methods:**

PubMed, Embase, Web of Science, and WANFAN were searched for studies performed prior to January 31, 2018, to collect data for our research. Meta-analysis was performed using RevMan 5.3 or STATA 14.0.

**Results:**

In total, 18 studies that met our criteria were included. Overall or HWE subgroup analysis of individuals with this polymorphism revealed that in terms of CP susceptibility, there was a significant difference between case groups and control groups in the A allele versus C allele model (OR = 1.38, 95% CI = 1.17–1.64 or OR = 1.38, 95% CI = 1.12–1.70), in the AA versus CC+CA model (OR = 1.49, 95% CI =1.06–2.10 or OR = 1.42, 95% CI = 1.13–1.78), and in the CC versus CA+AA model (OR = 0.69, 95% CI = 0.51–0.92 or OR = 0.68, 95% CI = 0.49–0.93); subgroup analysis based on a nonsmoking population also displayed significance in the A allele versus C allele model (OR = 1.43, 95% CI = 1.15–1.79) and CC versus CA+AA model (OR = 0.62, 95% CI = 0.44–0.87). For this polymorphisms and AgP susceptibility, our analyses revealed a significant association in both the A allele versus C allele model (OR = 1.29, 95% CI = 1.01–1.63) and the AA versus CC+CA model (OR = 1.93, 95% CI = 1.30–2.89); subgroup analysis based on Caucasian or nonsmoking populations showed significant differences in the AA versus CC+CA model (OR = 6.29, 95% CI = 1.78–22.21 or OR = 3.24, 95% CI = 1.59–6.61).

**Conclusions:**

IL-10-592 (-590, -597) A allele and the associated AA genotype may be risk factors for the onset of CP or AgP—particularly for the AA genotype and the increased risk of AgP in Caucasian or nonsmoking populations. Conversely, the CC genotype may act as a protective factor against the onset of CP.

## 1. Introduction

It was previously reported that specific genetic factors can account for as much as 50% of the overall onset risks of periodontal disease [1, 2]. Recently, gene polymorphisms in some cytokines such as interleukin- (IL-) 1, IL-6, TNF-*α*, IL-8, and IL-10 have been shown to play a vital role in the occurrence of both chronic and aggressive periodontitis (CP and AgP) immune pathogenesis [3–7].

IL-10 is an anti-inflammatory cytokine and a B lymphocyte proliferation factor, having pleiotropic effects on both immune regulation and inflammation [8]. It can stimulate the production of protective antibodies or autoimmunity proteins, while also being capable of downregulating the expression of proinflammatory cytokines, such as IL-1, IL-2, and IL-6 [9, 10]. The IL-10 gene is located on chromosome 1q31-32 [11], and at its promoter region, there are three polymorphic loci, -1082 (-1087) A>G (rs1800896), -819 (-824) C>T (rs1800871), and -592 (-590, -597) C>A (rs1800872), that have been identified [12]. The A allele at the -592 (-590, -597) position is known to have a negative regulatory function [13].

Meta-analyses exclusively on the association of IL-10-592 (-590, -597) C>A polymorphisms with periodontitis have not been extensively reported on, though two papers by Zhong et al. [14] and Albuquerque et al. [15] briefly touched on the topic as part of a larger body of work. Zhong et al. [14] used meta-analysis in 2012 to find out that the IL-10-592 (-590, -597) A allele and AA genotype were significantly associated with an increased CP risk, while the A allele alone was significantly associated with CP risk in people of Caucasian ethnicity. However, Zhong et al. [14] did not investigate the association of IL-10-592 (-590, -597) gene polymorphisms with AgP risk. A meta-analysis by Albuquerque et al. [15] found out that the IL-10-592 (-590, -597) A allele was significantly associated with susceptibility to both CP and AgP. This was especially true for susceptibility to CP in Caucasian individuals. Albuquerque et al. [15] also found out that the CC genotype at this locus was associated with resistance to CP onset in Caucasian population. Importantly, the two meta-analyses only included studies conducted before 2009 and thus only involved in six studies that included 624 CP cases and 623 controls [16–21] and five studies that included 411 CP cases, 97 AgP cases, and 442 controls [16–20], respectively. Since that time, there have been another 12 studies [22–33] reporting the association between IL-10-592 (-590, -597) C>A polymorphisms and periodontitis susceptibility, though the findings have been inconsistent. Therefore, in the present meta-analysis, we include a total of 18 studies [16–33] to further identify the contributions of IL-10-592 (-590, -597) gene variations to periodontitis (CP/AgP) susceptibility in a larger number and range of patients.

## 2. Materials and Methods

The meta-analysis was performed in accordance with the PRISMA-P (preferred reporting items for systematic reviews and meta-analysis protocols) statement which was recommended for the establishment of a systematic review and meta-analysis [34].

### 2.1. Focused Question

Is there an association between IL-10-592 (-590, -597) gene variations and CP or AgP?

### 2.2. Inclusion Criteria

The inclusion criteria that published studies need to meet to be included in the meta-analysis were as follows: (1) case-control studies; (2) the case groups consisted of patients diagnosed with CP or AgP, and the control groups consisted of periodontally healthy individuals; (3) IL-10-592 (-590, -597) C>A polymorphisms were detected, and sufficient data regarding genotype distributions were provided for the calculation of odds ratio (ORs) and corresponding 95% confidence intervals (95% CIs); and (4) studies with no repeated data. Studies that did not meet each of these criteria were excluded from the meta-analysis.

### 2.3. Search Strategy

A systematic literature search for studies published up to January 31, 2018, was performed using the electronic databases, PubMed, Embase, Web of Science, and WANFAN. In addition, the reference lists of the selected manuscripts and related reviews were also manually screened for comprehensive results. The search strategies were presented as follows.

#### 2.3.1. Search Strategies

*(1) PubMed*. Periodontal Diseases or Periodontitis or Periodontics or periodontal disease (title/abstract) or periodontitis (title/abstract) or periodontal pocket (title/abstract) or periodontal tissue (title/abstract) and Interleukin-10 or Interleukin-10 (title/abstract) or title/abstract (all fields) or IL-10 (title/abstract) or IL 10 (title/abstract) or Interleukin 10 (title/abstract) and polymorphism, genetic or genetic variation or polymorphism (title/abstract) or polymorphisms (title/abstract) or genetic variation (title/abstract) or rs1800872 (title/abstract).

*(2) Embase*. Periodontal disease/exp or periodontitis/exp or periodontics/exp or periodontal disease: ab, ti or periodontitis: ab, ti or periodontal pocket: ab, ti or periodontal tissue: ab, ti and Interleukin 10′/exp or Interleukin 10: ab, ti or IL10: ab, ti or rs1800872: ab, ti and DNA polymorphism/exp or genetic variability/exp or polymorphism: ab, ti or polymorphisms: ab, ti or genetic variation: ab, ti.

*(3) Web of Science*.

Number 1

TS = periodontal disease or

TS = periodontitis or TS = periodontal pocket or TS = periodontal tissue

Number 2

TS = interleukin-10 or TS = IL-10 or TS = interleukin 10 or TS = IL 10

Number 3

TS = polymorphism or

TS = polymorphisms or TS = genetic variation

Number 1 and Number 2 and Number 3

*(4) WANFAN*. Keywords in Chinese were used for the systematic search as follows: periodontal diseases, periodontitis, IL-10, interleukin-10, polymorphism, and the combined phrases.

### 2.4. Study Selection and Data Extraction

**Figure 1:**
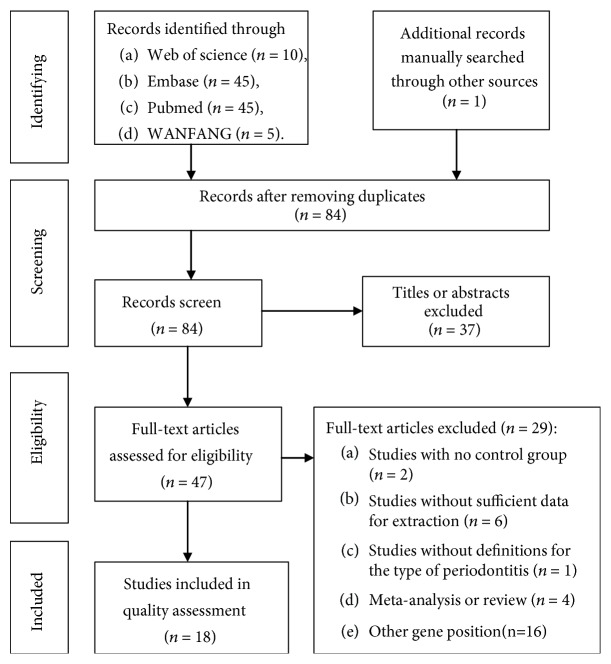
Screening process of the included studies.

For study selection, duplicate studies or datasets were firstly removed from the included titles using EndNote software. Then titles and abstracts of the remaining results were screened, followed by full-text paper screening according to the inclusion criteria described above (Figure 1). The results were screened by two authors independently, and a third author (JL Song) was consulted if any discrepancies existed. The following characteristics were extracted from the included studies by two authors independently, and discrepancies were resolved through discussion as follows: (1) the name of the first author and year of publication, (2) country (or district) and ethnicity of study participants, (3) group size, (4) smoking status, (5) gender ratio comparability, (6) type of controls, (7) genotype distribution, and (8) the Hardy–Weinberg equilibrium (HWE) results for the controls.

### 2.5. Quality Assessment

The Newcastle-Ottawa Scale (NOS) was used to assess the quality of the included case-control studies, which was performed by two authors independently. The composition of NOS includes three sections for consideration, which were “Selection” (0–4 points), “Comparability” (0–2 points), and “Exposure” (0–3 points). For the “Comparability” chapter, smoking status, age, and sex were selected as the main confounding factors to be matched in the present study. If two out of three factors were matched, one point (asterisk) was scored; if all three factors were matched, two points were assigned. The final scores were calculated ranging from 0 to 9. Studies with scores of 0–3, 4–6, and 7–9 points were considered of low, moderate, and high quality, respectively [6, 35].

### 2.6. Data Analysis

The ORs and 95% CIs were calculated to evaluate the association between IL-10-592 (-590, -597) polymorphisms and susceptibility to chronic or aggressive periodontitis. Heterogeneity between studies was estimated by *χ* ^2^ and *I* ^2^. An *I* ^2^ > 50% or *P* < 0.05 was considered to have significant heterogeneity. Next, the Mantel–Haenszel random effects model was used to assure the pooled efficiency. Otherwise, the Mantel–Haenszel fixed-effects model was used. The following three genetic models were applied for the meta-analyses of the IL-10-592 (-590, -597) polymorphisms: (1) allele comparison, (2) the dominant model, and (3) the recessive model. In addition, the *χ* ^2^ test method was used to assess the Hardy–Weinberg equilibrium (HWE) for the control groups. Subgroup analyses were conducted based on ethnicity, HWE fulfillment, and smoking status. The potential publication bias was measured by Begg's and Egger's linear regression tests. A publication bias was considered significant if *P* < 0.05. All statistical analyses were processed using the statistical software RevMan (version 5.3; The Nordic Cochrane Centre, The Cochrane Collaboration, Copenhagen, Denmark, 2014) or STATA 14.0.

## 3. Results

### 3.1. Characteristics and Quality Assessment of Included Studies

A total of 18 articles were included in the current meta-analysis on the gene IL-10-592 (-590, -597) C>A (rs1800872) polymorphisms. The selection process for including publication articles is presented in the flowchart (Figure 1). The characteristics of included studies are presented in Table 1. As shown in Table 1, 18 studies encompassing 2191 cases (1903 CP cases and 288 AgP cases) and 1975 controls were involved in the analysis of associations between IL-10-592 (-590, -597) C>A polymorphisms and the occurrence of either CP or AgP. Caucasian individuals alone were the focus of seven studies. Claudino et al. [18] and Garlet et al. [23] recruited a mixed population for their study, containing both Caucasian individuals and African-Americans as research subjects. Eleven studies documented nonsmokers in CP and AgP cases and controls. Allelic and genotypic data of IL-10-592 (-590, -597) C>A are shown in Table 1. With the exception of studies by Garlet et al. [23], Gorgun et al. [29], Toker et al. [30, 33], and Moudi et al. [32], the genotype distributions in the control groups of the other studies were consistent with HWE.

The scores of NOS ranged from 4 to 8. Nine studies were considered to be of high quality [16, 17, 22–24, 29, 30, 32, 33], and the other 9 studies were classed as moderate quality [18–21, 25–28, 31] (Table 2, e-Table 1).

### 3.2. Meta-Analysis Results of Association between IL-10-592 (-590, -597) C>A Polymorphisms and the Risk of CP

**Figure 2:**
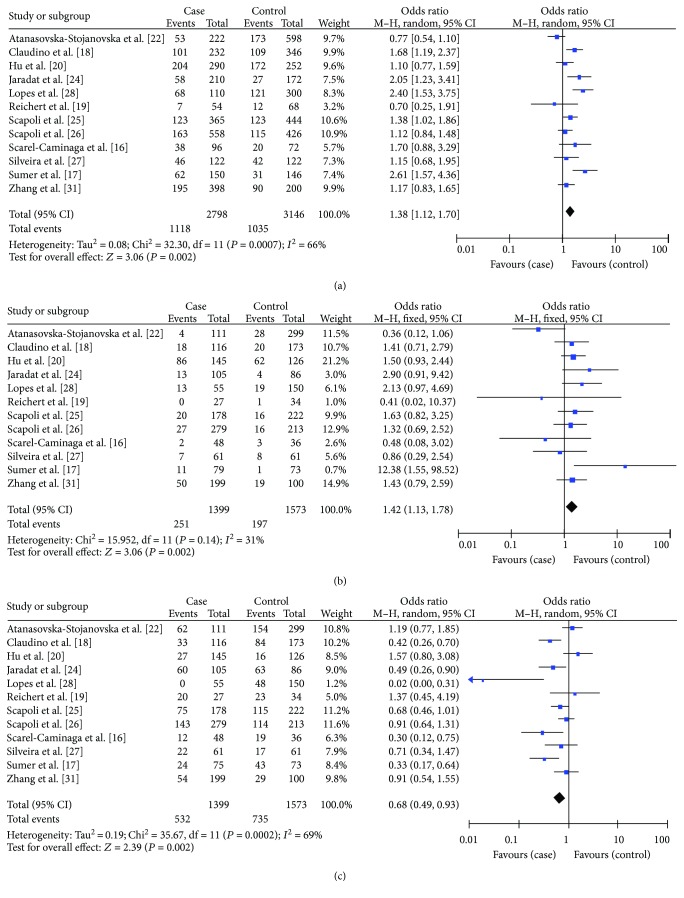
Forest plot of the association between IL-10-592 (-590, -597) C>A polymorphisms and chronic periodontitis by excluding the studies deviated from HWE in the control. (a) A allele versus C allele model. (b) AA versus AC+CC model. (c) CC versus AC+AA model.

Our literature search yielded 16 viable studies that had been conducted on the association between IL-10-592 (-590, -597) C>A polymorphisms and the risk of acquiring CP. The results are summarized in Table 3. We found out that there were significant associations in the A allele versus C allele model (OR = 1.38, 95% CI = 1.17–1.64), in the AA versus CC+CA model (recessive model; OR = 1.49, 95% CI = 1.06–2.10), and in the CC versus CA+AA model (dominant model; OR = 0.69, 95% CI = 0.51–0.92) (Table 3). When stratified by HWE, significant differences were also found in the A allele versus C allele model (OR = 1.38, 95% CI = 1.12–1.70), in the AA versus CC+CA model (OR = 1.42, 95% CI = 1.13–1.78), and in the CC versus CA+AA model (OR = 0.68, 95% CI = 0.49–0.93). In this analysis, we excluded the studies by Garlet et al. [23], Toker et al. [30, 33], and Moudi et al. [32], as genotype distributions in their control groups were deviated from HWE (Table 3, Figures 2(a)–2(c)).

**Figure 3:**
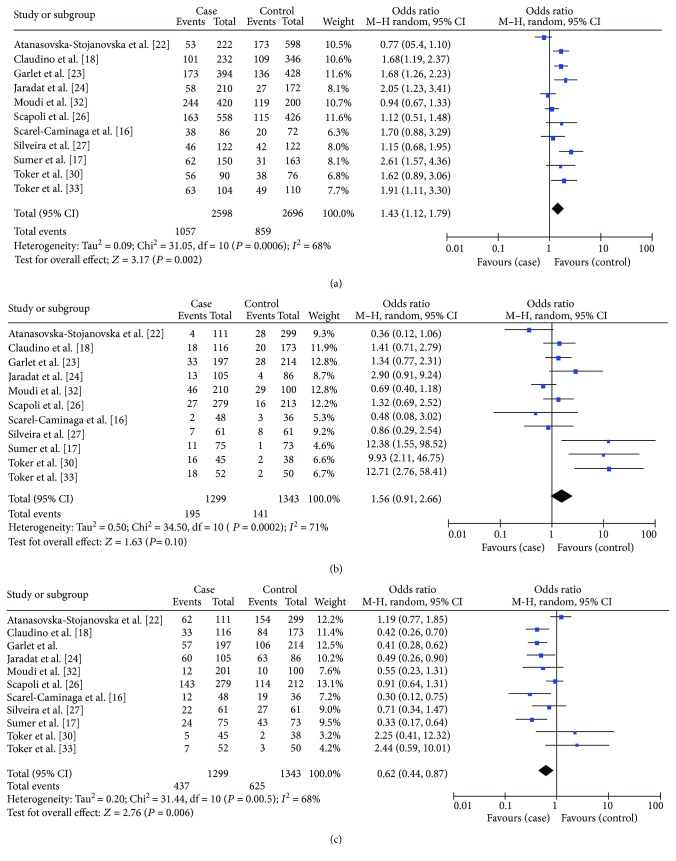
Forest plot of the association between IL-10-592 (-590, -597) C>A polymorphisms and chronic periodontitis in nonsmoking population. (a) A allele versus C allele model. (b) AA versus AC+CC model. (c) CC versus AC+AA model.

When we considered only the Caucasian subgroup, we found no significant differences in the A allele versus C allele model, in the AA versus CC+CA model, or in the CC versus CA+AA model (Table 3). Conversely, in the nonsmoker subgroup, we did find significant differences in both the A allele versus C allele model (OR = 1.43, 95% CI = 1.15–1.79) and CC versus CA+AA model (OR = 0.62, 95% CI = 0.44–0.87) (Table 3, Figures 3(a) and 3(c)).

### 3.3. Meta-Analysis Results of Association between IL-10-592 (-590, -597) C>A Polymorphisms and the Risk of AgP

**Figure 4:**
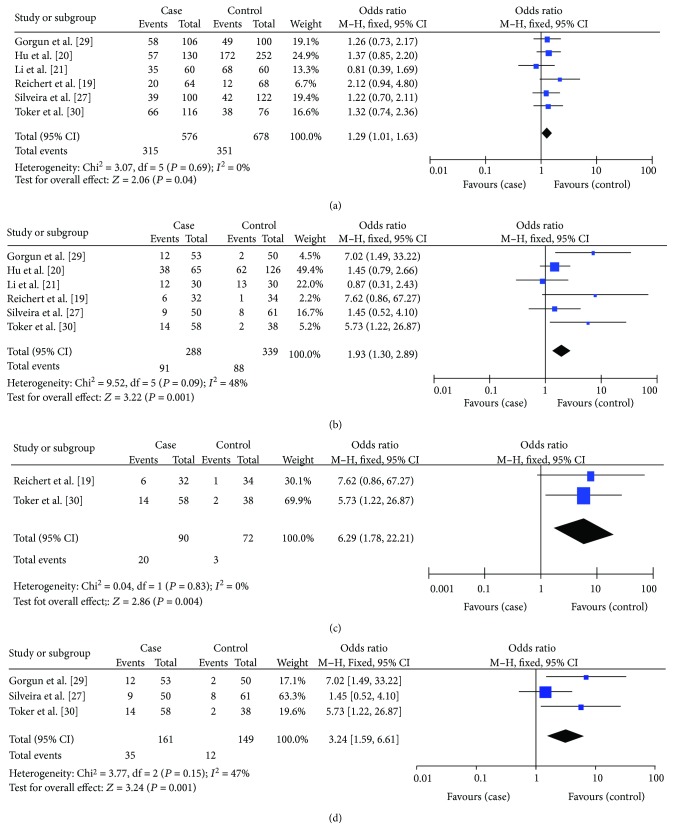
Forest plot of the association between IL-10-592 (-590, -597) A>C polymorphisms and aggressive periodontitis. (a) A allele versus C allele model. (b) AA versus AC+CC model. (c) AA versus AC+CC model in Caucasian population. (d) AA versus AC+CC model in nonsmoking population.

Six studies were involved, comprising 288 cases and 399 controls. The results are summarized in Table 3. The overall analyses of these studies yielded significant estimates in the A allele versus C allele model (OR = 1.29, 95% CI = 1.01–1.63) and in the AA versus CC+CA model (OR = 1.93, 95% CI = 1.30–2.89) (Table 3, Figures 4(a) and 4(b)) but no significant estimates in the CC versus CA+AA model (Table 3). Subgroup analyses by ethnicity showed significant trends in the Caucasian population in the A allele versus C allele model (OR = 1.55, 95% CI = 0.97–2.48) and significant differences in the AA versus CC+CA (OR = 6.29, 95% CI = 1.78–22.21) (Table 3, Figure 4(c)), but no significant estimates were found in the CC versus CA+AA model (Table 3). The nonsmoker subgroup analysis did, however, reveal significant differences in the AA versus CC+CA model (OR = 3.24, 95% CI = 1.59–6.61) (Table 3, Figure 4(d)).

### 3.4. Sensitivity Analysis

To assess the effect of an individual dataset on pooled ORs, a sensitivity analysis was performed through the sequential omission of each study. The results suggested that no single study greatly influenced the pooled estimations under any of the three genetic models for CP (e-Tables 2–4).

### 3.5. Publication Bias

Egger's test proved that there was no significant publication bias except HWE fulfillment or Caucasian population subgroup analysis in the CP versus controls allele comparison (Table 3). There was also not any obvious evidence of publication bias by Egger's test in overall and subgroup analysis in the AgP versus controls alleles and genotypes comparison (Table 3).

## 4. Discussion

### 4.1. Summary of Evidence

The present meta-analysis included 18 studies with 2191 cases and 1975 controls. There are some conflicting results among the 18 studies included, which may be the result of variations in individual study characteristics, including the ethnic populations surveyed, different sample sizes, and key confounding variables such as smoking status. Therefore, the present meta-analysis increases the likelihood of identifying true correlations by further systematizing the existing information.

Investigations of the correlations between IL-10-592 (-590, -597) C>A polymorphisms and CP risk suggested that IL-10-592 (-590, -597) A allele and AA genotype may increase the risk of CP, while CC genotype provides increased protection against the risk of the disease. These results were expected because 11 out of 16 individual studies included in our meta-analysis presented these trends in their populations. Furthermore, sensitivity analysis revealed no quantitative changes for the interstudy heterogeneity, suggesting that these results were stable and trustworthy.

It was demonstrated that the associations between genetic polymorphisms and certain diseases varied with different geographical regions and ethnic groups [6]. Therefore, we also investigated the contributions of ethnicity to the risk of disease. Our results suggested that the IL-10-592 (-590, -597) A allele and AA or CC genotypes were not associated with any increased risk that Caucasian individuals may have toward CP. These results varied from the meta-analysis results reported by Zhong et al. [14] and Albuquerque et al. [15] in which they found out that the A allele might increase the risk for CP, and Albuquerque et al. [15] found out the CC genotype might resist the risk among Caucasians. We hypothesize that part of the reason for this variation is that our meta-analysis included four additional studies that yielded inconsistent results [22, 25, 26, 30].

For IL-10-592 (-590, -597) C>A polymorphisms and AgP, our meta-analysis results indicated that the A allele may confer a relative increase in the risk for developing AgP, especially in Caucasian individuals, as described in the meta-analysis results reported by Albuquerque et al. [15]. The same conclusions can be drawn in relation to the comparison AA versus AC/CC genotypes, which is in contrast to the results reported by Albuquerque et al. [15].

Smoking may increase the risk of periodontitis onset [36–38], so we performed nonsmoker subgroup analyses in order to account for the effect of smoking status on the risk of developing CP or AgP. Our findings confirmed that there was a significant association between the occurrence of the A allele and CP individuals, just as there was with the CC genotype and healthy individuals. These results further showed that IL-10-592 (-590, -597) A allele may be the susceptible factor for the onset of CP while CC genotype may be the protective effect against CP occurrence (OR = 0.62, 95% CI = 0.44–0.87). Among the related 11 studies, five of them, reported by Jaradat et al. [24], Sumer et al. [17], Claudino et al. [18], Garlet et al. [23], and Toker et al. [33], showed that the A allele occurred significantly more frequently in the CP population than in the control group, respectively, while another four studies reported by Scapoli et al. [26], Scarel-Caminaga et al. [16], Silveira et al. [27], and Toker et al. [30] also described these trends, respectively. Studies reported by Sumer et al. [17], Claudino et al. [18], Scarel-Caminaga et al. [16], Garlet et al. [23], and Jaradat et al. [24] indicated that the CC genotype at the -592 or -597 positions occurred significantly more frequently in the control group than in the CP group. Additional three studies reported by Moudi et al. [32], Scapoli et al. [26], and Silveira et al. [27] previously present these trends. Therefore, we obtained positive results that were consistent with most other studies. The AA genotype in 3 studies conducted by Sumer et al. [17] and Toker et al. [30, 33] occurred significantly more frequently in the CP group than in the control group. Four studies, those conducted by Scapoli et al. [26], Jaradat et al. [24], Garlet et al. [23], and Claudino et al. [18] presented these trends, but four additional studies carried out by Atanasovska-Stojanovska et al. [22], Moudi et al. [32], Scarel-Caminaga et al. [16], and Silveira et al. [27] did not show these trends. Our present meta-analysis also did not show significantly positive results, suggesting that the AA genotype might not be the risk for CP in a nonsmoking population. However, the AA genotype might increase the risk for AgP onset in nonsmoker population because our meta-analysis results showed a threefold greater difference between the case and control groups under the AA versus AC+CC model for the AgP risk. These results were partially expected, as three related studies performed by Silveira et al. [27], Gorgun et al. [29], and Toker et al. [30] all showed the risk trend.

### 4.2. Strengths and Limitations

This meta-analysis has several strengths, including an unrestricted search process (including grey literature), duplicate review procedures for the search, sensitivity analysis, and assessments of the risk of bias and the quality of literature. But this meta-analysis has some limitations. As we know, interstudy heterogeneity and publication bias are main limitations associated with meta-analyses. Heterogeneity can be caused by many factors such as race, sample sizes, smoking habits, and deviations of allele distributions from the HWE [6]. There was obvious heterogeneity in the overall analysis involving CP individuals. We removed the studies from our analysis that deviated from HWE in the controls and performed the analysis again. Using these refined parameters, we still found that the results displayed higher heterogeneity in the A allele versus C allele model, as well as in the CC versus AC+AA model. Subgroup analysis based on Caucasian race or nonsmoking population also displayed higher heterogeneity. Further analysis found out that in the A allele versus C allele model, there was observed heterogeneity in the Atanasovska-Stojanovska et al. study [22], whose control sample sizes was larger, and from the Lopes et al. study [28], in which the pooled OR values were more than two, indicating over two times increased susceptibility to CP. When we concurrently removed the above two studies, the *I* ^2^ value decreased to less than 50%. In the CC versus AC+AA model, the main heterogeneity may have resulted from inclusion of the Atanasovska-Stojanovska et al. study [22], which had larger sample sizes in the control group, the Lopes et al. study [28] with zero CC individual in the case group, the Hu et al. study [20] with differential result, and the Scapoli et al. study [26], which had larger sample sizes in its case group. When these studies were removed, the *I* ^2^ value also decreased to less than 50%.

Another factor that we considered in our analysis was publication bias. Publication bias stems from the fact that positive results are much more readily published by journals, whereas negative results tend to be poorly received by journals and are collectively known as “grey literature” [6, 14, 15]. In the present study, we used Egger's test to probe the occurrence of publication bias, and the results indicated apparent publication bias in HWE fulfillment, as well as in the Caucasian population subgroup analysis in the CP versus controls allele comparison, which may have distorted our present results. We observed the funnel plot asymmetry of the two contrasts and found Atanasovska-Stojanovska et al. study [22], which contained a larger sample size, and Reichert et al. study [19], which comprised a smaller sample size, caused the publication bias. After excluding these two studies, the *P* value of Egger's test increased to over 0.05. However, the corresponding pooled OR values were not substantially altered without the publication bias.

Although a broad search in four different databases was used to find studies for inclusion in our meta-analysis, it is impossible to confirm that all available studies addressing the relationship between IL-10-592 (-590, -597) C>A polymorphisms and periodontitis were included, presenting another major limitation of the meta-analysis.

### 4.3. Implications for Clinical Practice

Three common single nucleotide polymorphisms (SNPs) in the IL-10 gene promoter (-1082 A>G, -819 C>T, and -592 C>A) show strong linkage disequilibrium and form two common haplotypes, designated as [ATA] and [GCC]. The [ATA] haplotype has been associated with decreased synthesis of IL-10 and is frequently associated with periodontitis [18]. Owing to the linkage disequilibrium, the presence of those haplotypes can be fully determined by the analysis of the IL-10-592 (-590, -597) C>A polymorphism, in which the occurrence of the A allele indicates the presence of the [ATA] haplotype [18]. Our findings showed that IL-10-592 (-590, -597) A allele or AA genotype existed extensively in CP and AgP populations, especially in Caucasian AgP populations. Therefore, the IL-10-592 (-590, -597) A allele or AA genotype may be a putative biomarker for the diagnosis of CP and AgP. We suggest that when patients are initially diagnosed with periodontitis clinically, testing for IL-10-592 (-590, -597) polymorphisms may be helpful in confirming diagnosis. Doctors and dentists may also routinely consider monitoring the IL-10-592 (-590, -597) A allele or AA genotype in healthy population to prevent the occurrence of CP and AgP by recommending prophylactic measures. Such prophylactic measures include no smoking, regularly seeing dentists for professional examination, removal of microbial biofilm, and so forth, brushing teeth twice daily for 2 minutes with a soft toothbrush, brushing the tongue, cleaning the interdental spaces with interdental aids (such as floss or interproximal brushes), using a fluoride toothpaste, and having a balanced diet, among others [39].

## 5. Conclusion

Even considering the limitations of this study, the present meta-analysis supported the hypothesis that IL-10-592 (-590, -597) C>A polymorphisms may be associated with CP and AgP susceptibility. We not only identified that the IL-10-592 (-590, -597) A alleles and AA genotypes may be a risk factor for the development of CP and AgP but also found out that the IL-10-592 (-590, -597) CC genotype may play a protective role in preventing CP. It is noteworthy that the AA genotype was found to be more closely tied to the risk of AgP in Caucasian and nonsmoker population. Thus, IL-10-592 (-590, -597) A alleles or AA genotypes may be a putative biomarker for diagnosing CP and AgP. Large-scale studies to further validate our findings should be performed in the future.

## Figures and Tables

**Table 1:** Characteristics of the included studies on association of IL-10-592 (-590, -597) C>A gene polymorphisms with periodontitis.

Author (year)	Population	Study type	Cases/controls	Smoking status	Control type	Gender	Quality (NOS)	Locus of SNPs
Scarel-Caminaga et al. (2004) [16]	Brazilian Caucasian	Case-control	48/36	No smoke	HC	Matched	7/9	-592
Sumer et al. (2007) [17]	Turkish Caucasian	Case-control	75/73	No smoke	CC	Matched	8/9	-597
Claudino et al. (2008) [18]	Brazilian (mixed)	Case-control	116/173	No smoke	CC	Not Matched	6/9	-592
Reichert et al. (2008) [19]	German Caucasian	Case-control	59/34	Mixed	CC	Matched	6/9	-590
Hu et al. (2009) [20]	Taiwanese Han	Case-control	210/126	Mixed	HC	Matched	5/9	-592
Li et al. (2009) [21]	Chinese Han	Case-control	30/30	Unknown	HC	Matched	6/9	-592
Atanasovska-Stojanovska et al. (2012) [22]	Macedonian Caucasian	Case-control	111/299	No smoke	HC	Matched	7/9	-592
Garlet et al. (2012) [23]	Brazilian (mixed)	Case-control	197/214	No smoke	HC	Matched	7/9	-592
Jaradat et al. (2012) [24]	Jordanian	Case-control	190/86	No smoke	CC	Matched	7/9	-597
Scapoli et al. (2012) [25]	Italian Caucasian	Case-control	182/230	Mixed	Unknown	Unknown	5/9	-592
Scapoli et al. (2015) [26]	Italian Caucasian	Case-control	182/230	No smoke	Unknown	Matched	6/9	-592
Silveira et al. (2016) [27]	Brazilian	Case-control	111/61	No smoke	HC	Matched	6/9	-592
Gorgun et al. (2017) [29]	Turkish	Case-control	53/50	No smoke	HC	Matched	7/9	-597
Lopes et al. (2017) [28]	Brazilian	Case-control	55/150	Unknown	HC	Matched	4/9	-592
Toker et al. (2017) [30]	Turkish Caucasian	Case-control	103/38	No smoke	HC	Matched	6/9	-597
Zhang et al. (2017) [31]	Chinese Uygur	Case-control	200/100	Unknown	CC	Matched	7/9	-597
Moudi et al. (2018) [32]	Iranian	Case-control	210/100	No smoke	HC	Matched	7/9	-592
Toker et al. (2018) [33]	Turkish	Case-control	52/50	No smoke	HC	Matched	7/9	-592

HC/CC: hospital/community control; NOS: Newcastle-Ottawa scale; SNPs: single-nucleotide polymorphisms.

**Table 2:** Distribution of IL-10-592 (-590, -597) C>A genotypes and allele frequency among periodontitis patients and control subjects.

First author	Year	Cases (CP/AgP)	Controls	Genotype distribution						Allele distribution				*P* for HWE (control)
				Cases			Controls			Cases		Controls		
				CC	AC	AA	CC	AC	AA	C	A	C	A	
Scarel-Caminaga [16]	2004	48 (CP)	36	12	34	2	19	14	3	58	38	52	20	0.85
Sumer [17]	2007	75 (CP)	73	24	40	11	43	29	1	88	62	115	31	0.11
Claudino [18]	2008	116 (CP)	173	33	65	18	84	69	20	131	101	237	109	0.32
Reichert [19]	2008	27 (CP)	34	20	7	0	23	10	1	47	7	56	12	0.94
		32 (AgP)		18	8	6				44	20			
Hu [20]	2009	145 (CP)	126	27	32	86	16	48	62	86	204	80	172	0.17
		65 (AgP)		6	21	38				33	97			
Li [21]	2009	30 (AgP)	30	7	11	12	5	12	13	25	35	22	38	0.275
Atanasovska-Stojanovska [22]	2012	111 (CP)	299	62	45	4	154	117	28	169	53	425	173	0.40
Scapoli [25]	2012	178 (CP)	222	75	83	20	115	91	16	233	123	321	123	0.73
Garlet [23]	2012	197 (CP)	214	57	107	33	106	80	28	221	173	292	136	0.04
Jaradat [24]	2012	105 (CP)	86	60	32	13	63	19	4	152	58	145	27	0.13
Scapoli [26]	2015	279 (CP)	213	143	109	27	114	83	16	395	163	311	115	0.87
Silveira [27]	2016	61 (CP)	61	22	32	7	27	26	8	76	46	80	42	0.66
		50 (AgP)		20	21	9				61	39			
Gorgun [29]	2017	53 (AgP)	50	7	34	12	3	45	2	48	58	51	49	<0.001
Lopes [28]	2017	55 (CP)	150	0	42	13	48	83	19	42	68	179	121	0.07
Toker [30]	2017	45 (CP)	38	5	24	16	2	34	2	34	56	38	38	<0.001
		50 (AgP)		6	38	14				50	66			
Zhang [31]	2017	199 (CP)	100	54	95	50	29	52	19	203	195	110	90	0.96
Moudi [32]	2018	210 (CP)	100	12	152	46	10	61	29	176	214	81	119	0.008
Toker [33]	2018	52 (CP)	50	7	27	18	3	45	2	41	63	61	49	<0.001

CP/AgP: chronic/aggressive periodontitis; HWE: Hardy–Weinberg equilibrium fulfillment.

**Table 3:** IL-10-592 (-590, -597) C>A polymorphism and periodontitis.

Genetic model	Total/subgroup	Number of studies	Cases/controls	OR (*P* value)	95% CI	*I* ^2^, % (*P* value^a^)	Egger (*P* value)	Model of meta-analysis
IL-10-592 (-590, -597) C>A polymorphism and chronic periodontitis								
A versus C	Total	16	1903/1975	1.38 (<0.001)	1.17–1.64	62.1 (0.001)	0.071	Random
	Total for HWE^b^	12	1418/1580	1.38 (0.002)	1.12–1.70	65.9 (<0.001)	0.017	Random
	Caucasian	7	763/915	1.31 (0.088)	0.96–1.77	68.4 (0.004)	0.047	Random
	Nonsmoker	11	1299/1343	1.43 (0.002)	1.15–1.79	68.0 (<0.001)	0.294	Random
AA versus CC+CA	Total	16	1903/1975	1.49 (0.02)	1.06–2.10	59.4 (0.001)	0.243	Random
	Total for HWE	12	1399/1573	1.42 (0.002)	1.13–1.78	31.0 (0.14)	0.234	Fixed
	Caucasian	7	763/915	1.50 (0.33)	0.66–3.43	68.0 (0.005)	0.647	Random
	Nonsmoker	11	1299/1343	1.56 (0.10)	0.91–2.66	71.0 (<0.001)	0.993	Random
CC versus AA+CA	Total	16	1903/1975	0.69 (0.01)	0.51–0.92	68.0 (<0.001)	0.208	Random
	Total for HWE	12	1399/1573	0.68 (0.02)	0.49–0.93	69.0 (<0.001)	0.149	Random
	Caucasian	7	763/915	0.74 (0.15)	0.50–1.11	66.2 (0.007)	0.676	Random
	Nonsmoker	11	1299/1343	0.62 (0.006)	0.44–0.87	68.0 (<0.001)	0.450	Random
IL-10-592 (-590, -597) C>A polymorphism and aggressive periodontitis								
A versus C	Total	6	288/399	1.29 (0.04)	1.01–1.63	0.0 (0.69)	0.28	Fixed
	Caucasian	2	90/72	1.55 (0.07)	0.97–2.48	0.0 (0.35)	—	Fixed
	Nonsmoker	3	161/149	1.26 (0.157)	0.92–1.74	0.0 (0.98)	—	Fixed
AA versus CC+CA	Total	6	288/399	1.93 (0.001)	1.30–2.89	47.5 (0.09)	—	Fixed
	Caucasian	2	90/72	6.29 (0.004)	1.78–22.21	0.0 (0.83)	—	Fixed
	Nonsmoker	3	161/149	3.24 (0.001)	1.59–6.61	47.0 (0.15)	—	Fixed
CC versus AA+CA	Total	6	288/399	0.98 (0.91)	0.64–1.49	0.0 (0.52)	0.43	Fixed
	Caucasian	2	90/72	0.88 (0.76)	0.39–2.01	34.0 (0.22)	—	Fixed
	Nonsmoker	3	161/149	1.19 (0.58)	0.65–2.17	5.6 (0.35)	—	Fixed

OR: odds ratio; CI: confidence interval. ^a^ *P* value for heterogeneity. ^b^Hardy–Weinberg equilibrium fulfillment.

## Supplementary Materials

Supplementary Materials e-Table 1: Newcastle-Ottawa quality assessment scores for the studies included in the meta-analysis. e-Table 2: sensitivity analysis^∗^ for the A allele versus C allele model and CP risk. e-Table 3: sensitivity analysis^∗^ for the AA versus AC+CC model and CP risk. e-Table 4: sensitivity analysis^∗^ for the CC versus AA+AC model and CP risk.
