# Supplementary material for: Contribution of Interleukin-10-592 (-590, -597) C>A Polymorphisms to Periodontitis Susceptibility: An Updated Meta-Analysis Based on 18 Case-Control Studies
Source: Dis Markers. 2018 Sep 19;2018:2645963. doi: 10.1155/2018/2645963 (PMC6169208; doi:10.1155/2018/2645963)
Supplement: Supplementary Materials — e-Table 1: Newcastle-Ottawa quality assessment scores for the studies included in the meta-analysis. e-Table 2: sensitivity analysis∗ for the A allele versus C allele model and CP risk. e-Table 3: sensitivity analysis∗ for the AA versus AC+CC model and CP risk. e-Table 4: sensitivity analysis∗ for the CC versus AA+AC model and CP risk. [file 2645963.f1.pdf]

e-TABLE 1. Newcastle-Ottawa quality assessment scores for the studies included in the meta-analysis.

Author(Year)	Selection	Comparability	Exposure	NOS	Quality
Scarel-Caminage (2004)[16]	★★★	★★	★★	7/9	High
Sumer(2007)[17]	★★★★	★★	★★	8/9	High
Claudino(2008)[18]	★★★	★	★★	6/9	Moderate
Reichert(2008)[19]	★★★★	-	★★	6/9	Moderate
Hu(2009)[20]	★★★	-	★★	5/9	Moderate
Li(2009)[21]	★★★	★	★★	6/9	Moderate
Atanasovska-Stojanovska(2012)[22]	★★★	★★	★★	7/9	High
Garlet(2012)[23]	★★★	★★	★★	7/9	High
Jaradat(2012)[24]	★★★	★★	★★	7/9	High
Scapoli(2012)[25]	★★★	-	★★	5/9	Moderate
Scapoli(2015)[26]	★★★	★	★★	6/9	Moderate
Silveira(2016)[27]	★★★	★	★★	6/9	Moderate
Gorgun(2017)[29]	★★★	★★	★★	7/9	High
Lopes(2017)[28]	★★	-	★★	4/9	Moderate
Toker(2017)[30]	★★★	★★	★★	7/9	High
Zhang(2017)[31]	★★★★	-	★★	6/9	Moderate
Moudi(2018)[32]	★★★	★★	★★	7/9	High
Toker(2018)[33]	★★★	★★	★★	7/9	High

e-TABLE 2. Senstivity analysis* for the A allele vs. C allele model and CP risk.

Author(Year)	OR	95%CI	*P*	I^2	*P* for I^2
All authors	1.38	1.17-1.64	0.002	62	0.0005
Omitted Atanasovska-Stojanovska(2012)[22]	1.44	1.23-1.69	<0.00001	52	0.009
Omitted Claudino(2008)[18]	1.36	1.14-1.63	0.0007	63	0.0005
Omitted Garlet(2012)[23]	1.36	1.14-1.63	0.0008	62	0.0008
Omitted Hu(2009)[20]	1.42	1.18-1.69	0.0002	64	0.0005
Omitted Jaradat(2012)[24]	1.35	1.14-1.61	0.0006	62	0.0008
Omitted Lopes(2017)[28]	1.33	1.13-1.57	0.0006	57	0.03
Omitted Moudi(2018)[32]	1.43	1.20-1.70	<0.0001	60	0.001
Omitted Reichert(2008)[19]	1.41	1.19-1.69	<0.0001	63	0.0005
Omitted Scapoli(2012)[25]	1.39	1.15-1.67	0.0005	65	0.0003
Omitted Scapoli(2015)[26]	1.41	1.18-1.69	0.0002	63	0.0006
Omitted Scarel-Caminage (2004)[16]	1.37	1.15-1.63	0.0004	64	0.0004
Omitted Silveira(2016)[27]	1.40	1.17-1.67	0.0002	64	0.0003
Omitted Sumer(2007)[17]	1.33	1.13-1.57	0.0005	57	0.003
Omitted Toker(2017)[30]	1.37	1.15-1.64	0.0004	64	0.0003
Omitted Toker(2018)[33]	1.37	1.15-1.64	0.0004	64	0.0003
Omitted Zhang(2017)[31]	1.40	1.17-1.68	0.0003	64	0.0007

* The sensitivity analysis was performed through the sequential omission of each study.

e-TABLE 3. Senstivity analysis* for the A A vs. AC +CC model and CP risk.

Author(Year)	OR	95%CI	*P*	I^2%	*P* for I^2
All authors	1.49	1.06- 2.10	0.02	59	0.001
Omitted Atanasovska-Stojanovska(2012)[22]	1.60	1.15- 2.23	0.005	55	0.006
Omitted Claudino(2008)[18]	1.51	1.04- 2.20	0.03	62	0.0007
Omitted Garlet(2012)[23]	1.53	1.04- 2.24	0.03	62	0.0007
Omitted Hu(2009)[20]	1.51	1.03- 2.22	0.04	62	0.0008
Omitted Jaradat(2012)[24]	1.44	1.02- 2.05	0.04	60	0.001
Omitted Lopes(2017)[28]	1.46	1.01- 2.09	0.04	61	0.001
Omitted Moudi(2018)[32]	1.61	[1.14-2.26]	0.006	53	0.008
Omitted Reichert(2008)[19]	1.52	1.07- 2.14	0.02	62	0.0009
Omitted Scapoli(2012)[25]	1.49	1.03- 2.17	0.03	62	0.0008
Omitted Scapoli(2015)[26]	1.53	1.05- 2.22	0.03	62	0.0008
Omitted Scarel-Caminage (2004)[16]	1.54	1.09-2.18	0.01	61	0.001
Omitted Silveira(2016)[27]	1.55	1.08- 2.21	0.02	61	0.001
Omitted Sumer(2007)[17]	1.42	1.02- 1.97	0.04	57	0.003
Omitted Toker(2017)[30]	1.38	[1.00- 1.91]	0.05	54	0.007
Omitted Toker(2018)[33]	1.37	1.00-1.86	0.05	51	0.01
Omitted Zhang(2017)[31]	1.52	1.04-2.21	0.04	62	0.0008

* The sensitivity analysis was performed through the sequential omission of each study.

e-TABLE 4. Senstivity analysis* for the CC vs. AA + AC model and CP risk.

Author(Year)	OR	95%CI	*P*	I^2%	*P* for I^2
All authors	0.69	0.51-0.92	0.01	68	<0.0001
Omitted Atanasovska-Stojanovska(2012)[22]	0.65	0.48- 0.87	0.004	65	0.0002
Omitted Claudino(2008)[18]	0.72	0.53- 0.97	0.03	68	<0.0001
Omitted Garlet(2012)[23]	0.72	0.53- 0.97	0.03	65	0.0002
Omitted Hu(2009)[20]	0.64	0.48- 0.86	0.003	66	0.0002
Omitted Jaradat(2012)[24]	0.70	0.52-0.96	0.02	70	<0.0001
Omitted Lopes(2017)[28]	0.71	0.54- 0.93	0.01	65	0.0002
Omitted Moudi(2018)[32]	0.69	0.51-0.94	0.02	70	<0.0001
Omitted Reichert(2008)[19]	0.67	0.49- 0.89	0.007	69	<0.0001
Omitted Scapoli(2012)[25]	0.69	0.50- 0.95	0.02	70	<0.0001
Omitted Scapoli(2015)[26]	0.67	0.48- 0.92	0.01	68	<0.0001
Omitted Scarel-Caminage (2004)[16]	0.72	0.53-0.96	0.03	68	<0.0001
Omitted Silveira(2016)[27]	0.68	0.50- 0.93	0.02	70	<0.0001
Omitted Sumer(2007)[17]	0.72	0.54- 0.97	0.03	67	<0.0001
Omitted Toker(2017)[30]	0.67	0.50- 0.89	0.006	69	<0.0001
Omitted Toker(2018)[33]	0.66	0.49-0.88	0.005	68	<0.0001
Omitted Zhang(2017)[31]	0.67	0.49-0.91	0.01	70	<0.0001

* The sensitivity analysis was performed through the sequential omission of each study.
